# Bypassing birthing centres for child birth: a community-based study in rural Chitwan Nepal

**DOI:** 10.1186/s12913-016-1848-x

**Published:** 2016-10-21

**Authors:** Rajani Shah

**Affiliations:** Shree Medical and Technical College, Bharatpur-12, Chitwan, Nepal

**Keywords:** Bypassing, Birthing centre, Rural area, Nepal, Equitable access, Quality service

## Abstract

**Background:**

Child delivery in a health facility is important to reduce maternal mortality. Bypassing nearby birthing facility to deliver at a hospital is common in developing countries including Nepal. Very little is known about the extent and determinants of bypassing the birthing centres in Nepal. This study measures the status of bypassing, characteristics of bypassers and their reasons for bypassing.

**Methods:**

A community-based cross-sectional study was carried out in six rural village development committees of Chitwan district of Nepal. Structured interviews were conducted with 263 mothers who had given birth at a health facility and whose nearest facility was a birthing centre. Descriptive statistics, univariate and multivariable logistic regression analysis were performed.

**Results:**

More than half of the mothers had bypassed the nearer birthing centres to deliver at hospital. Living in plain area [aOR: 2.467; 95 % CI: 1.005–6.058], higher wealth index [aOR: 4.981; 95 % CI: 2.482–9.999], advantaged caste/ethnicity [aOR: 2.172; 95 % CI: 1.153–4.089], older age [aOR: 2.222; 95 % CI: 1.050–4.703] and first birth [aOR: 2.032; 95 % CI: 1.060–3.894] were associated with higher likelihood of bypassing. Among the reasons of bypassing as reported by the bypassers, lack of operation, video x-ray, and blood test facilities were the most common ones, followed by the lack of medicines/drugs and equipment, lack of skilled service provider, and inadequate physical facilities, among others.

**Conclusions:**

Quality of service at the birthing centres needs to be given a high consideration to increase their use as well as to ensure an equitable access to the quality care by all.

**Electronic supplementary material:**

The online version of this article (doi:10.1186/s12913-016-1848-x) contains supplementary material, which is available to authorized users.

## Background

Among the 289,000 maternal deaths that happen worldwide, 99 % occur in developing countries [1]. Although maternal mortality ratio (MMR) has declined in Nepal, it still stands at the second highest level among the South Asian countries [1]. Availability, access, and utilization of safe motherhood service along with the quality of the service are important in reducing the maternal deaths [2]. In particular, availability of birthing facilities is one of the critical strategies to reduce maternal mortality in developing countries [3].

Globally health centres led by midwives that provide basic emergency obstetric care serve as the ‘first level contact of care’ for childbirth close to the settings women live in and the birthing culture and rituals of the community. Referring complicated cases to hospital for comprehensive emergency obstetric care saves mothers and newborns lives [4–6]. Birthing centres should be staffed by midwives trained to manage normal pregnancies, child birth and the postnatal period, and to identify, manage and refer mother and newborn if complications arise [7].

Government of Nepal has been promoting expansion of birthing centres in a phase-wise manner in health facility of rural areas where access to health service is difficult for women [8]. The birthing centres are being attached to health posts and health centres of rural areas. At least two midwives are posted at a centre to provide the service for 24-hours [9]. However, referral system is not instituted properly in Nepal [10].

**Fig. 1:**
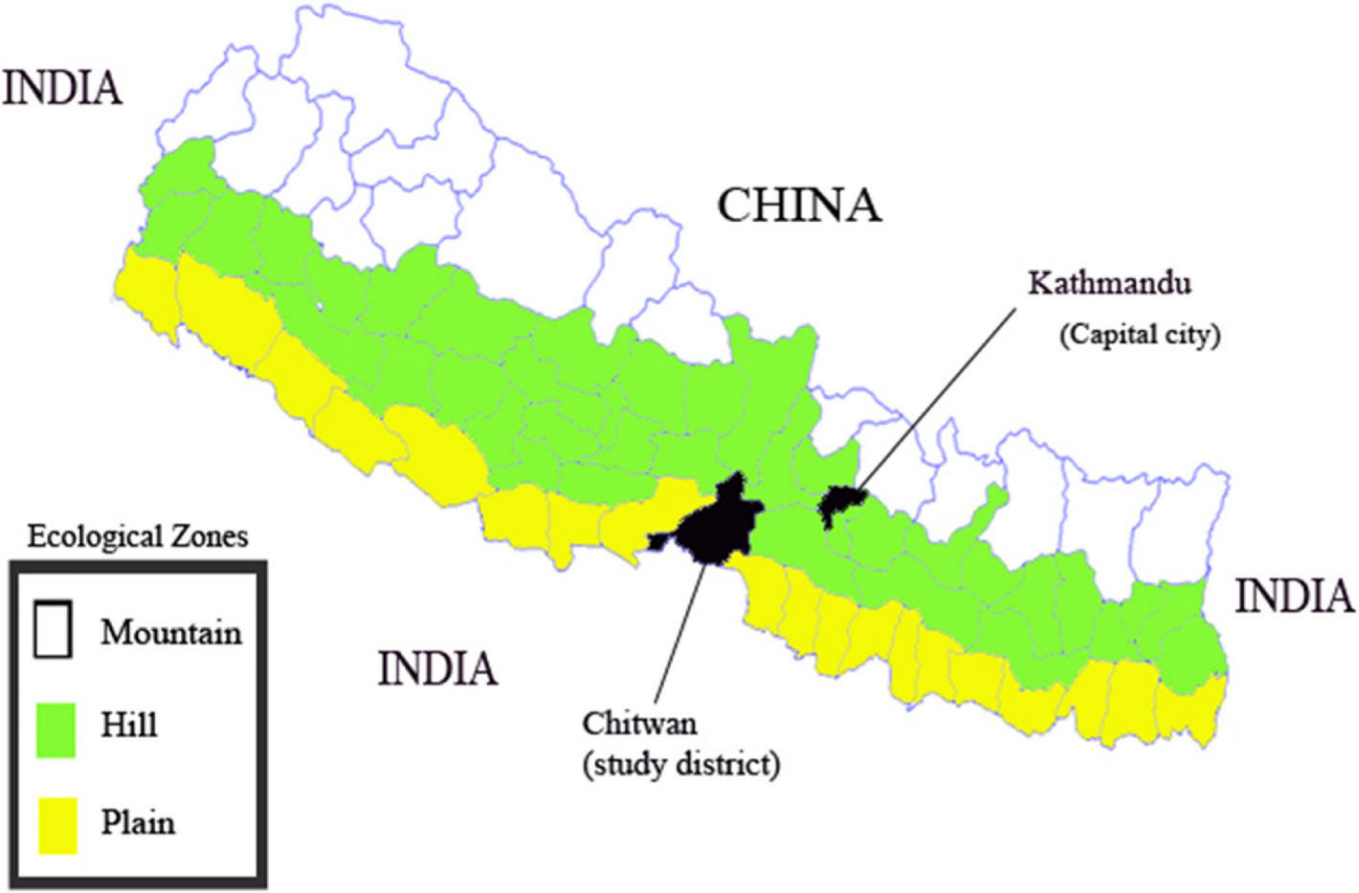
Ecological zones of Nepal and study district (Chitwan)

Utilization of the health services differs largely between the people of advantaged (Brahman/Chhetri and advantaged Janjati) and disadvantaged caste/ethnicity (disadvantaged Janjati and Dalits), with the latter one having less access to health services due to their poorer socio-economic development [11]. Similarly, among the three ecological zones of Nepal (Fig. 1), utilization of the childbirth service is lower in hill and mountain than in plain [12] due to difficulty in reaching to health facility owing to lack of transportation and difficult topography.

Despite the delivery service being free, a significant amount of out-of-pocket expenses incur accessing delivery service at a health facility, particularly in transportation, medicines, medical tests and X-rays, staying in maternity waiting homes and supplies [13]. Though the service at private health facility would cost more on drugs, tests and treatment [14], in Nepal accessing the service of private institutions for childbirth increased from 1.3 % in 1996 to 10.4 % in 2011 [15]. However, nearly three quarters (73.65 %) of the health facility deliveries happen in the government sector with only 20.28 % in private sector [16].

Rural areas in developing countries have usually health facilities with poorer quality service [17, 18]. Bypassing primary health care facilities takes place to get better quality service of hospital in terms of competency of service provider, availability of drugs and equipments, and quality of care [13]. A significant number of people tend to bypass the nearest health facilities to access the service including birthing facility of a hospital at a further distance [19–23].

A very limited information is available on bypassing birthing centres in Nepal [24]. Therefore, this study examines the status of bypassing birthing centre, characteristics of the women who bypass, and their reasons of bypassing.

## Methods

### Study setting

Chitwan district has a total population of 579,948 with 66 % rural population. People with various caste/ethnicity reside in the district including Brahman, Chhetri, Tharu, Tamang, Magar, Gurung, Newar, Chepang, Kami, Magar, Kumal, Darai, Sarki, Musalman, etc. More than three quarters (77 %) of population have upto five years schooling [25]. Although Chitwan is considered a plain district, some of its parts are hilly (Fig. 1). At the time of data collection, the district was divided into two municipalities and 36 village development committees (VDCs), with nine hill and the rest of the others plain VDCs. Recently, some VDCs have been merged to make other new municipalities. All municipalities and VDCs of the district are connected with roads except one hill VDC.

Though there are several private hospitals and nursing homes in the district [25], the major three referral hospitals comprise of one governmental and two teaching hospitals located in the district headquarter. The rural areas have been served by the 18 birthing centres at health posts and primary health care centres [25]. In this study, six rural VDCs were selected on the basis of lower percentage of health facility delivery than the average of the district. Then all eligible women of six randomly selected wards out of nine from each of the selected VDCs were included. The details of sampling process has been explained somewhere else [26].

### Study design, study participants and sample size

**Fig. 2:**
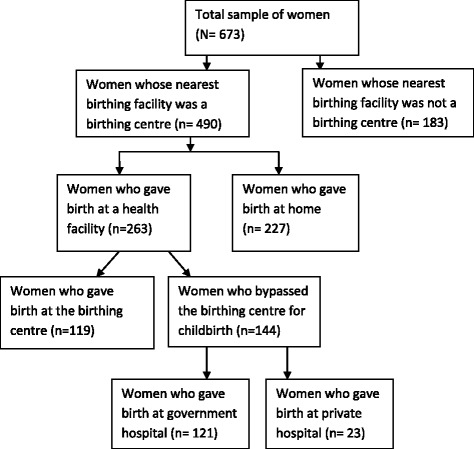
Selection of study participants

A community-based cross-sectional study was carried out including 673 women who had given birth during April 21, 2012 and April 20, 2013 in the selected study areas. Among these, for 490 women birthing centre was the nearest birthing facility, while it was a hospital for the remaining 183 women. This study was limited to 263 women whose nearest birthing facility was a birthing centre and had given birth at a health facility. Of the 263 women, 119 gave birth at the nearest birthing centre, whereas 144 bypassed and went to a hospital (Fig. 2).

### Data collection

First a list of mothers who had given birth during the study period was prepared from the records of female community health volunteers (FCHVs). Records of mothers were cross-checked from both pregnancy and delivery registers maintained by the FCHVs under community-based newborn care program [10]. Additionally, inquiry was made in the communities for any other potential missed cases of birth.

Data collection was done between April 26, 2013 and June 30, 2013. Face-to-face interviews were conducted using structured questionnaire (Additional file 1) making household visits. Nepal Health Research Council granted ethical approval to the study (registration number 21/2012). The purpose of the study was explained and informed consent from the participants or from their mother-in-law/husband/other guardian for those who were below the legal age was obtained before the data collection. Voluntary participation of the participants was ensured throughout the study by advising them that they had the right to refuse to participate or to withdraw their participation at any time without prejudice. Personal identifiers were removed from the datasets before analysis. Students of Bachelor of Public Health and Masters of Sociology with previous experience of data collection were recruited as data enumerators. They were provided with orientation on the data collection and involved in pretesting of the questionnaire [27].

### Statistical analysis

Outcome variable, i.e., bypasser status, has been categorized as bypassers and non-bypassers. Bypassers are women who bypassed the nearest birthing centre and delivered at hospital (coded 1), while non-bypassers are those who gave birth at the nearest birthing centre (coded 0). Eight independent variables have been included in the study. Place of residence: The place of residence of the women was categorized into ‘hill’ and ‘plain’ area. Caste/Ethnicity: The caste/ethnicity has been categorized as ‘advantaged’ and ‘disadvantaged’ caste/ethnicity. The advantaged group includes the upper caste (Brahman/Chhetri) and advantaged Janjati (Newar and Gurung). The disadvantaged caste/ethnicity includes the disadvantaged Janjati and lower caste (Dalits) [11, 28]. Educational status: The number of schooling year has been categorized as ‘no schooling’ and ‘primary (upto grade five) and above’. Wealth index: A relative wealth index was constructed by principal component analysis based on the household ownership of assets [29]. The women were categorized into two groups- with ‘lower’ and ‘higher’ wealth index. Age: The completed age in years at the time of childbirth has been categorized into ‘15–24 years’ and ‘25 or more years’. Birth order: The order of the recent birth has been categorized as ‘1^st’^ and ‘2^nd^ or more’. Complication during delivery: Experience of complication during the recent childbirth has been categorized as ‘yes’ if the women experienced complication and ‘no’ if the women did not experience complication. Antenatal care (ANC): It is the number of ANC visit a woman had during the recent pregnancy. It has been categorized as ‘<4’ and ‘4 or more’.

Data entry was done in Epi data version 3.1 [30] and data analysis was carried out in Statistical Package for the Social Sciences version 16.0 [31]. Descriptive statistics was used to identify the distribution of bypassers and non-bypassers across different independent variables. Logistic regression analysis was used [32] for univariate and multivariable analysis. The multivariable logistic regression analysis was performed including all the independent variables simultaneously in a model.

## Results

### Status of bypassing

Among the 263 women whose nearest birthing facility was a birthing centre and had health facility delivery, 119 (45 %) gave birth in the birthing centre, while 144 (55 %) bypassed and went to a hospital at a further distance for the birth. Among the bypassers, a large majority (84 %) went to government hospital, while the remaining 16 % went to a private hospital.

### Characteristics of women associated with bypassing

Table 1 shows the distribution of characteristics of women by the status of bypassing. Nearly two third of women (63 %) living in plain area bypassed, while it was only 21 % among women residing in hill. Nearly three quarters of women from advantaged caste/ethnicity bypassed in comparison to 44 % among disadvantaged caste/ethnicity. Similarly, about three quarters of women with higher wealth index bypassed compared to only 23 % of those with lower wealth index. The likelihood of bypassing was higher among women with more education. A slightly higher percentage of women with age 25 and more years bypassed than the women in the age group 15–24 years. A higher proportion of women having four or more ANC visit bypassed the nearest birthing facility compared to the women having less than four ANC visits (62 % versus 40 %). A larger proportion of women both with first parity and experiencing complication during birth bypassed than the women with two or more births and having no complication respectively.

**Table 1:** Distribution of characteristics of women by the status of bypassing

Characteristics	Status of bypassing		
	Not Bypass	Bypass	Total
Place of residence			
Plain	78 (37)	133 (63)	211
Hill	41 (79)	11 (21)	52
Caste/ethnicity			
Disadvantaged	91 (56)	76 (44)	167
Advantaged	28 (29)	68 (71)	96
Wealth index			
Lower	69 (78)	20 (22)	89
Higher	50 (29)	124 (71)	174
Educational status			
No schooling	34 (71)	14 (29)	48
Primary and above	85 (40)	130 (61)	215
Age group			
15–24 years	88 (48)	97 (52)	185
25 and more years	31 (40)	47 (60)	78
Birth order			
1^st^	54 (39)	84 (61)	138
2^nd^ and more	65 (52)	60 (48)	125
ANC number			
< 4	53 (60)	38 (40)	91
4 or more	66 (38)	106 (62)	172
Experience of complications during delivery			
No	105 (48)	114 (52)	219
Yes	14 (32)	30 (68)	44

Table 2 depicts that in univariate logistic regression analysis five variables, namely, living in plain area, advantaged caste/ethnicity, higher wealth index, first birth and less than 4 ANC visits were found to be statistically significantly associated with the higher odds of bypassing. Likewise, the multivariable analysis also showed five variables having statistically significant associations with bypassing. However, frequency of ANC visit lost its significance, while age of the women appeared to have an association. The women living in plain were more than 2 times [Adjusted odds ratio (aOR): 2.467; 95 % CI: 1.005–6.058] more likely to bypass than women in hill. Similarly, bypassing was more than 2 times [aOR: 2.172; 95 % CI: 1.153–4.089] higher also among women from advantaged caste/ethnicity compared to disadvantaged ones. Women with higher wealth index had about 5 times [aOR: 4.981; 95 % CI: 2.482–9.999] higher odds of bypassing. Likewise, the women with age group 25 and more years and giving first birth had also more than 2 times [aOR: 2.222; 95 % CI: 1.050–4.703 and [aOR: 2.032; 95 % CI: 1.060–3.894] greater likelihood of bypassing compared to younger women and for higher birth order respectively.

**Table 2:** Factors associated with bypassing

Variables	Unadjusted OR (95 % CI)	*P*-value (Crude)	Adjusted OR (95 % CI)	*P*-value (adjusted)
Place of residence				
Plain	**6.355 (3.088–13.081)**	<0.001	**2.467 (1.005–6.058)**	0.049
Hill	1.00		1.00	
Caste/ethnicity				
Disadvantaged	1.00		1.00	
Advantaged	**3.714 (1.882–7.330)**	<0.001	**2.172 (1.153–4.089)**	0.016
Wealth index				
Lower	1.00		1.00	
Higher	**8.556 (4.713–15.532)**	<0.001	**4.981 (2.482–9.999)**	<0.001
Educational status				
No schooling	1.00		1.00	
Primary and above	1.468 (0.666–3.236)	0.341	2.152 (0.921–5.029)	0.077
Age group				
15–24 years	1.00		1.00	
25 and more years	1.375 (0.804–2.354)	0.245	**2.222 (1.050–4.703)**	0.037
Birth order				
1^st^	**1.685 (1.033–2.750)**	0.037	**2.032 (1.060–3.894)**	0.033
2^nd^ and more	1.00		1.00	
ANC number				
< 4	**2.240 (1.335–3.759)**	0.002	0.908 (0.487–1.695)	0.762
4 or more	1.00		1.00	
Experience of complications during delivery				
No	1.00		1.00	
Yes	1.974 (0.992–3.925)	0.053	1.621 (0.846–3.107)	0.146
Nagelkerke R Square				0.360
Hosmer and Lemeshow Test				0.411

Bold font indicates statistical significance with *p* < 0.05

The Hosmer and Lemeshow test result shows that the data fit into the model well [33].

### Reasons of bypassing

Lack of operation /video x-ray/blood test facility at the nearest birthing centres was the most common reason (59 %) for bypassing, followed by lack of necessary equipments/drugs (43 %), lack of skilled health worker (42 %), inadequate physical facilities (36 %), low confidence in service provider (25 %), etc. (Table 3).

**Table 3:** Reasons of bypassing reported by bypassers (multiple response analysis)

Reasons of bypassing	Frequency (*n* = 144)	Percentage
Lack of operation/video x-ray/blood test facility	78	59
Lack of necessary drugs/equipments	57	43
Lack of skilled health worker	56	42
Inadequate physical facilities	48	36
Low confidence of health worker	33	25
Service provider not available	2	1.5
Others^a^	3	2.3
Don’t know	3	2.3

^a^On Saturday health post is closed/No knowledge of service/not open for 24 h

## Discussion

### Status of bypassing

The study found that though birthing centres were closer, more than half (55 %) of the women bypassed the centres and gave birth at a hospital. The bypassing status in the current study is higher than in rural Tanzania [23] where 42.2 % of women who had health facility delivery had bypassed the nearest birthing facility. Similarly, another study of rural Tanzania showed 41.8 % women bypassing the birthing service of the local primary care clinic [21]. The figure of bypassing in the current study is, however, lower than that of the previous study in Nepal which found 70.2 % of women having health facility delivery had bypassed birthing centres [24]. People generally tend to bypass the closer health facilities in favour of those with better quality of service. In a study in Nepal women rated birthing centres lowest in terms of adequacy of medical equipment and competency of health personnel compared to both public and private hospitals [34]. More wealthy and those having intrapartum complications were more likely to bypass the nearest birthing facility. Availability of operating facility, adequacy of medical supplies and equipment and competent health staff at the hospital were mentioned as the reasons of bypassing nearest birthing centres and choosing hospital to give birth [22].

### Factors associated with bypassing

Women living in plain area had higher odds of bypassing the nearer birthing centre than the women residing in hill. It could be because of the difficulty in travelling due to lack of roads and transportation in the hills.

In the current study women from advantaged caste/ethnicity were more likely to bypass compared to women from disadvantaged caste/ethnicity. A previous study in Nepal found no association of caste/ethnicity with bypassing [24]. The reason could lie in the types of caste/ethnicity and categorization of the variable. Unlike the previous study, the caste/ethnicity in the current study has been classified into only two categories, i.e., advantaged and disadvantaged. Barker et al also mentioned that access to safe motherhood services is largely influenced by caste/ethnicity [9].

Wealthier women were about five times more likely to bypass the birthing centre. Similar results were found in the previous studies in Nepal [24] and Tanzania [21]. Around a quarter of families in low- and middle-income countries have to borrow or/and sell their assets to pay for health care. Poorer families have to undergo more hardships [13, 35] which would make it difficult for them to access health services at a further distance due to costs related particularly to travelling further and staying at health facility.

Educational status of the women showed no effect on bypassing. This is consistent with the findings of a study in rural Tanzania where there was no association between bypassing and women having no schooling and some schooling [23].

Older women tended to bypass more than the younger ones. Similar results were observed in the previous study of Nepal where women aged 25 or more years had more than four times higher odds of bypassing than the younger women [24]. Likewise, in rural Tanzania 35 or more age group was associated with higher likelihood of bypassing than younger age [23]. Similarly, in Ghana a higher proportion of patients aged above 38 years had bypassed nearer health facility than the younger ones [36].

Bypassing was more common for first birth than the second or thereafter birth. Similar trends were observed in the previous studies of Nepal [24] and Tanzania [21].

Frequency of ANC visits showed no effect on bypassing. There are mixed results in the body of literature regarding the association between ANC visits and bypassing [22, 23]. Similarly, experience of complication during delivery was not associated with bypassing in the current study. The qualitative study carried out before the current study had found that the women who bypassed the nearest birthing centre had directly visited the farther hospital without having any complication. Similarly, in Ghana among the patients visiting an urban hospital 63% had bypassed local facility without experiencing any complication [36]. Having visited the hospital before was about three times more associated with bypassing the local facility. In the current study settings, the birthing centres had recently been in operation for only about two years preceding the survey. Access to health service depends not only on the distance from the home of a person but also upon the quality of health facilities [17]. As mentioned by Parkhurst et al the women might have bypassed the birthing centres due to the popularity of the hospitals they sought care and also because distance was not a problem to them due to the availability of the transportation facility in most of the study areas [37].

### Reasons of bypassing

In the current study, the most common reasons for bypassing were lack of operation /video x-ray/blood test facilities, lack of adequate drugs and equipment, and incompetent health worker at the nearer birthing centre. Consistent results were found in other studies conducted in rural Tanzania [19] and Nepal [24]. The study of Tanzania found lack of diagnostic facilities as the major reason, followed by lack of drugs, among others, whereas in the study of Nepal lack of operation facility was the most common reason followed by inadequate drugs and equipment, incompetent staff, etc. Similarly, unavailability of health worker was mentioned by a small percentage of women in all the three studies. The lack of confidence in health workers at birthing centres in this study could be due to lack of cadres appropriately trained as skilled birth attendants, as well as inadequate equipments.

The study has some potential limitations as well as strengths. A major limitation of the study is that bypassing by preference is not distinguished from referral by heath workers for women identified during pregnancy to be at higher risk of birthing complications (e.g. due to primigravida, grand multipara, pre-eclampsia, foetal distress, anaemia, previous history of complications etc.). This could partly explain some of the observed associations between bypassing and age and birth order. However, another study in Nepal found that 98 % of women attending referral hospitals bypass nearer birthing centres by preference [38], and this cannot explain the inequalities observed by caste and wealth groups. Secondly, since the study areas were selected on the basis of lower percentage of institutional delivery compared to the other VDCs of the district, the results may not be generalizable in all contexts. Nevertheless, the study provides information of the settings that are in priority with respect to equity in accessing quality service. Thirdly, there was a possibility of recall bias, however, limiting the study period to one year and most of the variables being socio-demographic characteristics of the women reduced the likelihood of the bias in the study. Lastly, other potential predictors were not included in the study, such as complications during pregnancy, previous pregnancies and birth, and actual quality of care at birthing centres.

## Conclusions

More than half of the women had bypassed the nearest birthing centre to give birth at a higher-level hospital with better quality of birthing service. Birthing centres have generally less competent health workers, inadequate drugs and equipment, poorer infrastructure and lack diagnostic facilities. Women residing in plain area, with higher wealth index, from advantaged caste/ethnicity, older women and giving first birth had higher likelihood of bypassing. It indicates inequity among women in accessing quality birthing service. Improvement in the quality of the service of rural birthing centres would ensure equitable access to the quality service by also women from hill, poorer, disadvantaged castes groups, younger women and women giving birth for second or more time. Improvement in the quality of the service at the local birthing centres would also reduce bypassing which would subsequently reduce financial burden to the family associated with travelling further among bypassers as well because people’s willingness to go further does not mean they are able to pay for the expenses [39].

## Additional file

Additional file 1: Survey tool used for interview with respondents. (DOCX 23 kb)

## Abbreviations

ANC: Antenatal care
aOR: Adjusted odds ratio
FCHV: Female Community Health Volunteer
MMR: Maternal mortality ratio
VDC: Village Development Committee
