# Supplementary material for: Bypassing birthing centres for child birth: a community-based study in rural Chitwan Nepal
Source: BMC Health Serv Res. 2016 Oct 21;16:597. doi: 10.1186/s12913-016-1848-x (PMC5073815; doi:10.1186/s12913-016-1848-x)
Supplement: Additional file 1: — Survey tool used for interview with respondents. (DOCX 23 kb) [file 12913_2016_1848_MOESM1_ESM.docx]

**Survey tool used for interview with respondents**

Form No............

Name of village development committee _________

Ward Number:

Name of the household head ________________

Name of interviewee __________

Interviewer's name __________ Date:

| **Q.N.** | **Characteristics** | **Questions** | **Response** | **Codes** |
| --- | --- | --- | --- | --- |
| **1.** | **Ethnicity** | What is your caste/ethnicity? | ............................................ |  |
| **2.** | **Educational level** | Have you ever attended school? | - Yes - No | 1  0 |
|  |  | *If had schooling*,  What is the highest grade you completed? | Number of schooling…….............. |  |
| **3. Economic status of the family** | | | |  |
| **3.1 Does your household have** | | | |  |
| Electricity supply? | | | 1. Yes 2. No |  |
| A radio? | | | 1. Yes 2. No |  |
| A television? | | | 1. Yes 2. No |  |
| A watch? | | | 1. Yes 2. No |  |
| A mobile telephone | | | 1. Yes 2. No |  |
| Other telephone | | | 1. Yes 2. No |  |
| A toilet | | | 1. Yes 2. No |  |
| Type of toilet | | | 1. Temporary 2. Permanent (water seal) |  |
| **3.2 Main material of the roof**  Observe and record | | | 1. Bamboo  2. Wood  3. Straw  4. Tin  5. Ceramic tiles  6. Cement  7. Other (specify)……… |  |
| **3.3 Main material of the exterior walls**  Observe and record | | | 1. Wood with mud  2. Bamboo with mud  3.Stone with mud  4. Plywood  5. Bricks  6. Cement blocks  7. Other (specify)………….. |  |
| **3.4 What is the main source of drinking water at your home?** | | | 1. Own piped water 2. Neighgour’s piped water 3. Tube well 4. Well 5. Public tap 6. Spring 7. Others (specify)………… |  |

| **4** | **Age** | How old were you when you gave birth to your last child? | …………………Years |  |
| --- | --- | --- | --- | --- |
| **5** | **Parity** | What is the birth order of this last delivered child? | ............................... |  |
|  |  | Live birth | ………………….. |  |
|  |  | Still birth | …………………… |  |
| **6** | **Number of antenatal care** | Did you receive antenatal care during the last pregnancy? | - Yes - No | 1  0 |
|  | If yes, | How many times did you receive antenatal care during this pregnancy? | Number of antenatal care……………..... |  |
| **7** | **Place of delivery** | Where did you give birth?    WRITE ALSO THE NAME OF THE PLACE.  ………………… | - Home - Government hospital - Primary health care   centre/Health post/Sub  health post   - Private   hospital/clinic/nursing  home   - Other (specify)………….. | 0  1  2  3 |
| **8** | **Nearest birthing facility** | Which birthing facility is nearer to your home?  (Write the specific name of the health facility)  ........................................... | - Government hospital - Primary health care centre - Health post/sub health post - Private   hospital/clinic/nursing  home   - Others (specify)......................... | 0  1  2  3 |
| **9** | **Distance to health facility** | How far is the nearest birthing facility from your home? | - Less than 30 minutes - 30 – 59 minutes - 60-120 minutes - More than two hours | 0  1  2  3 |
| **10** | *If gave birth at health institution somewhere else than the nearby birthing centre*,  **Reasons for bypassing birthing centre** | Why didn’t you deliver child at the birthing centre closer to your home? | - Health workers not available - No skilled health worker - Health workers not confident - No required drugs/equipment - No adequate physical facilities - No service for caesarean section if needed - No facility for ultrasound/blood testing - Others (specify)………….... | 1  2  3  4  5  6  7 |
| **11** | **Experience of complications** | Did you experience any kind of complication during the last three months of pregnancy, labour or delivery for this last birth? | -Yes  - No | 1  0 |
|  |  | During the last three months of pregnancy? | - Yes (specify)………… - No | 1  0 |
|  |  | During labour or delivery? | - Yes (specify)………… - No | 1  0 |
